# The efficacy and safety of post-stroke cognitive impairment therapies: an umbrella review

**DOI:** 10.3389/fphar.2023.1207075

**Published:** 2023-08-24

**Authors:** Yongbiao Li, Ruyi Cui, Shaobo Liu, Zhiping Qin, Wenjing Sun, Yong Cheng, Qingshan Liu

**Affiliations:** ^1^ Key Laboratory of Ethnomedicine of Ministry of Education, School of Pharmacy, Center on Translational Neuroscience, Minzu University of China, Beijing, China; ^2^ Institute of Chinese Materia Medica, China Academy of Chinese Medical Sciences, Beijing, China; ^3^ College of Life and Environmental Sciences, Minzu University of China, Beijing, China; ^4^ Institute of National Security, Minzu University of China, Beijing, China

**Keywords:** post-stroke cognitive impairment, clinical trial, systematic review, umbrella review, neurological functional

## Abstract

**Background:** Stroke survivors are at significantly increased risk of cognitive impairment, which affects patients’ independence of activities of daily living (ADLs), social engagement, and neurological function deficit. Many studies have been done to evaluate the efficacy and safety of post-stroke cognitive impairment (PSCI) treatment, and due to the largely inconsistent clinical data, there is a need to summarize and analyze the published clinical research data in this area.

**Objective:** An umbrella review was performed to evaluate the efficacy and safety of PSCI therapies.

**Methods:** Three independent authors searched for meta-analyses and systematic reviews on PubMed, the Cochrane Library, and the Web of Science to address this issue. We examined ADL and Barthel index (BI), Montreal Cognitive Assessment (MoCA), neurological function deficit as efficacy endpoints, and the incidence of adverse events as safety profiles.

**Results:** In all, 312 studies from 19 eligible publications were included in the umbrella review. The results showed that angiotensin-converting enzyme inhibitors (ACEI) and N-methyl-D-aspartate (NMDA) antagonists, cell therapies, acupuncture, and EGB76 can improve the MoCA and ADL, and the adverse effects were mild for the treatment of PSCI. Moreover, Vinpocetine, Oxiracetam, Citicoline, thrombolytic therapy, Actovegin, DL-3-n-Butylphthalide, and Nimodipine showed adverse events or low article quality in patients with PSCI. However, the research evidence is not exact and further research is needed.

**Conclusion:** Our study demonstrated that ACEI inhibitors (Donepezil) and NMDA antagonists (Memantine), EGB761, and acupuncture are the ADL and BI, MoCA, and neurological function deficit medication/therapy, respectively, for patients with PSCI.

**Clinical Trial Registration:** https://inplasy.com/inplasy-2022-11-0139/; Identifier: INPLASY2022110139.

## Introduction

Ischemic stroke seriously threatens human health and life, and is a common cardiovascular and cerebrovascular disease (Saini et al., 2021). According to the World Health Organization (WHO), the incidence of stroke has been increasing in recent years, and disability rates are very high. PSCI is a frequent complication after stroke, with a prevalence of 50%–70%, and effective treatment is needed to improve the prognosis of patients (Mijajlovic et al., 2017). Epidemiological studies have shown that stroke is the second leading cause of death in the world and the first fatal and disabling disease among the Chinese population. PSCI, including mild cognitive impairment and dementia, not only affects patients’ ability to do daily living but also hinders rehabilitation and exercise, increasing the economic and mental burden of family care. In addition, as little is known about the efficacy and safety of PSCI treatment in the recovery phase after stroke, the critical challenge in PSCI treatment is to determine the most effective way of current interventions.

There is some evidence supporting the notion that neurological deficits can be greatly improved by the use of recombinant tissue plasminogen activator (tPA) thrombolysis, ACEI inhibitors, and NMDA antagonists (Donepezil and galantamine) recommended by national guidelines for the treatment of PSCI (Ebihara et al., 2007; Liraz-Zaltsman et al., 2018; Yang et al., 2022). In addition, the neuroprotection, statins, and control of high-risk factors are recommended as secondary prevention of PSCI (Mijajlovic et al., 2017). Additionally, memantine and B1 and B2 bradykinin receptor agonists do not lead to significant improvement in PSCI cognition but provide overall functional benefits (Glass et al., 2020) (Martins, 2012). In addition, many clinical studies have shown that many other neuroprotective drugs improve cognitive impairment and are safe and effective (Zhang et al., 2014).

Much research has attempted to study the commonly used PSCI treatment methods. However, the results of these studies are still biased and contradictory (Wu et al., 2007). It is necessary to review the latest literature, delete duplicate or problematic studies, and then conduct a meta-analysis to obtain a pooled prevalence. Therefore, to draw a definitive conclusion and determine which commercially available therapies for PSCI patients are effective and safe, we have performed an umbrella review of the systematic reviews and meta-analyses of PSCI therapies through a comprehensive and updated literature search.

## Materials and methods

Our study conforms with the standard guidelines of Preferred Reporting Items for Systematic Reviews and Meta-analysis (Moher et al., 2009). The protocol for this review has been registered at INPLASY PROTOCOL (INPLASY2022110139).

### Search strategy and quality assessment

A systematic search of published peer-reviewed English-language literature was conducted using PubMed, Web of Science, and the Cochrane Library up until October 2022. The database search terms were as follows: (Post-stroke cognitive impairment/Post-stroke dementia) and (systematic review or meta-analysis) and clinical trial. We included meta-analyses and systematic reviews that determined the efficacy and safety of treatments in patients with PSCI. Inclusion criteria were: 1) articles written in English; 2) published systematic reviews or meta-analyses; 3) articles including any evaluation of clinical assessment scales for PSCI; and 4) articles published in peer-reviewed journals. Studies were excluded if 1) they were unpublished studies; 2) there were no necessary sample data; 3) patients were diagnosed with other PSCI; 4) the study reported insufficient details and other outcomes; and 5) there was a presence of risk of bias/study limitations.

We used the AMSTAR2 tool to evaluate systematic reviews and meta-analyses (De Santis et al., 2022). The methodological quality of the studies was determined by the percentage of the AMSTAR2 score. The percentage of the AMSTAR2 score was classified into 0%–15.8%, 15.8%–21.05%, and 21.05%–100%, indicating low quality, medium quality, and high quality, respectively.

We used keywords and filtered titles searching for related articles, and two review authors independently screened articles. These downloaded articles were screened by inclusion/exclusion criteria, and any irrelevant or duplicate articles were removed. Thereafter, we manually searched the reference lists from the selected literature for any other relevant studies that were not identified in the initial search. Finally, a full-text search was conducted to extract and analyze article data.

### Data extraction

According to the following criteria, three investigators (Yongbiao Li, Ruyi Cui, and Shaobao Liu) independently selected the trials that met the inclusion criteria. The main characteristics of the selected study were extracted and displayed in a table, including year of publication, study design, number of studies, and regimens for the treatment. We included results evaluating the efficacy of drugs in patients with at least one of the clinical assessment scales: 1) baseline mini-mental state examination (MMSE) scores; 2) the primary outcomes included global neurological deficit scores such as the National Institutes of Health Stroke Scale (NIHSS) score ≤1 and MoCA; 3) Alzheimer’s Disease Assessment Scale-Cognitive Subscale (ADAS-Cog); 4) dependence assessed by Clinical Global Impression of Change (CIBIC-plus or CGIC); 5) activities of ADL; 6) clinical effect, defined according to the nationally approved criteria, was divided into essentially recovered, significant improvement, improvement, no change, deterioration, and death (the first three categories were judged to be effective); 7) the secondary outcomes included: abilities of daily living (evaluated by BI), related hemorheology and lipid metabolism outcomes, and quality of life; and 8) incidence of adverse events (AE). The selection of assessments was extracted on study size, sample size, mean difference (Fixed, 95% CI) or odds ratio (Fixed, 95% CI), and heterogeneity (I^2^). A percentage of 0%–25% was classified as mild, 26%–50% was classified as moderate, and 51%–75% was classified as significant between-study heterogeneity. If I^2^ > 50%, a random-effects model was used for the analysis, or the data was analyzed on a fixed-effects model (Wang et al., 2016).

### Statistical analysis

Four clinical assessment scales were calculated using sample sizes and mean differences. The NIHSS/BI/SCORE was used to assess neurological status, and the patient’s behavioral symptoms were calculated using ADL/MMSE/ADAS. The clinical effects we focused on were divided into basic recovery, significant improvement, no change, and deterioration, as well as cognitive function scores and quality of life as an activity of daily living. Graphpad Prim 8 software was used for all clinical data analysis. Results are expressed as MD±SD (standard deviation). The incidence of adverse events was assessed and ORs were calculated. Therefore, the mean difference or odds ratio with 95% CI and *p* values were used to assess the effectiveness and safety of the study treatments.

## Results

**FIGURE 1:**
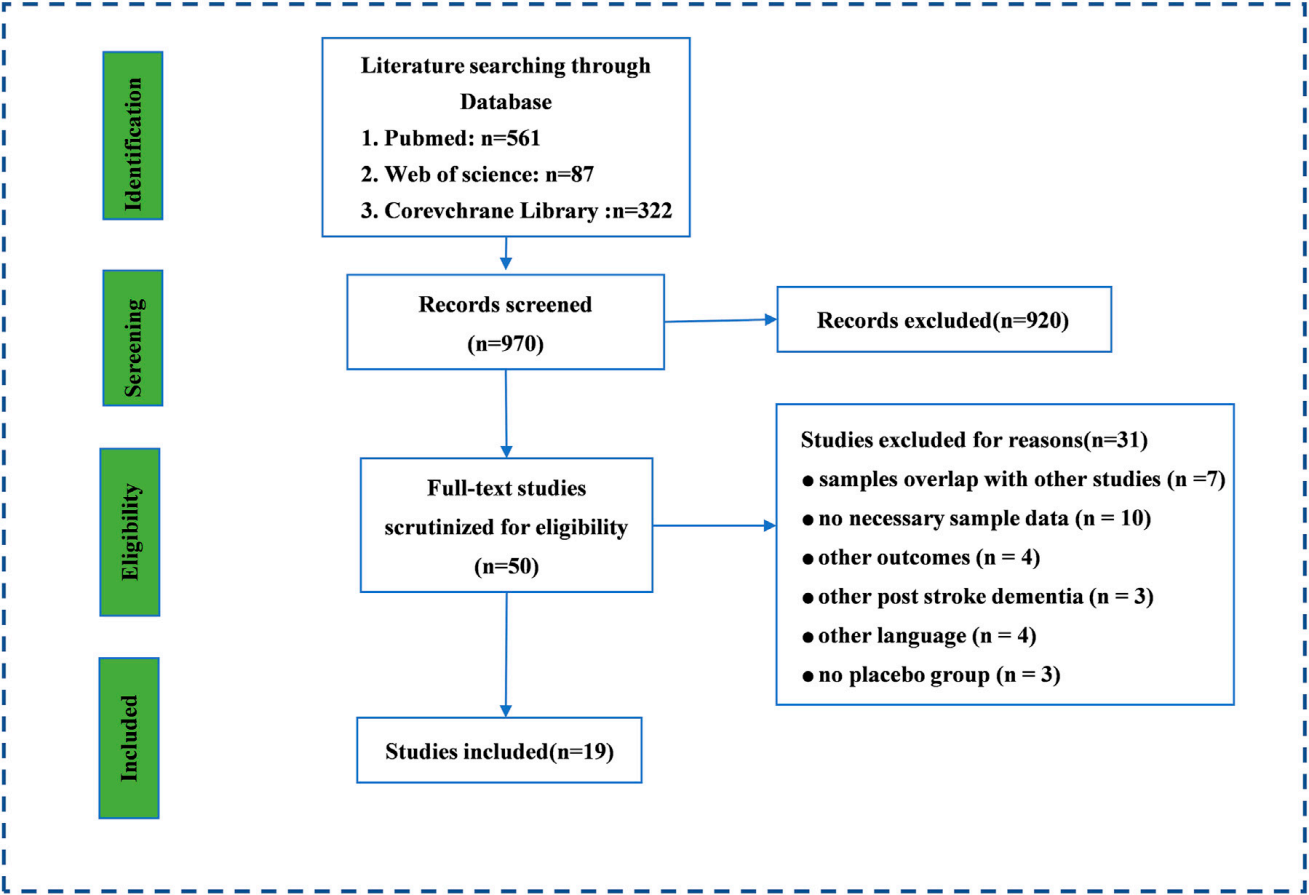
The search and screening process. Literature search and study selection. Through the initial search, we retrieved a total of 3,808 records from PubMed, Web of Science, and the Corevchrane Library. After examining the titles and abstracts, Through the initial search, 970 records was retrieved from PubMed, Web of science and Corevchrane Library. 50 studies were selected for further full-text scrutiny after examining the titles and abstracts. In all, 31 studies were excluded due to the following reasons: samples overlap with other studies (*n* = 7), no necessary sample data (*n* = 10), other outcomes (*n* = 4), other PSCI (*n* =3), other language (*n* = 4), no placebo group (*n* = 3).

Through the initial search, 970 records were retrieved from PubMed, Web of Science, and the Corevchrane Library. Then, 50 studies were selected for further full-text scrutiny after titles and abstracts were examined. In all, 31 studies were excluded due to the following reasons: samples overlap with other studies (*n* = 7), no necessary sample data (*n* = 10), other outcomes (*n* = 4), other PSCI (*n* = 3), not written in English (*n* = 4), and no placebo group (*n* = 3) (Figure 1). Thus, 19 studies were included in the umbrella review: Kim and Kang, (2020), Guekht et al. (2017), Kwon (2019), Huang et al. (2021), Tan (2015), Jin and Liu (2019), Shi et al. (2022), Lopez-Arrieta and Birks (2002), Fan (2021), Yi et al. (2020), Alvarez-Sabín. (2013), Huahui and Hehua (2015), Malykh and Sadaie (2010), Ming (2016), NanZhu et al. (2018), Wei (2020), You et al. (2019), Szatmári and Whitehouse (2009), Szatmari and Whitehouse (2003).

As shown in Table 1, a total of 312 clinical trials were included, with 19 drugs or drug combination therapies in the treatment groups. All studies were randomized controlled clinical trials, and the treatment duration ranged from 1 to 52 weeks. Among the included literature, there were 17 that were considered of high quality, 1 that was of moderate quality, and 1 that was of low quality.

**TABLE 1:** Description and the AMSTAR2 scores of included studies.

Study	Conditions	Studies included	Study duration (median, range)	Daily dose (median, range)	Outcomes	AMSTAR scores	Study quality
Kim and Kang, (2020)	Donepezil *versus* Placebo	7	5–25w	7.5 mg	1.MMSE,2.ADAS-cog, 3.CIBIC-plus orCGIC	10	high
Guekht et al. (2017)	Actovegin *versus* Placebo	9	52w	—	1.ADAS-cog, 2	9	high
Jin and Liu (2019)	Rivastigmine *versus* Placebo	12	18–24w	6 mg	1.ADAS-Cog, 2.MMSE, 3.ADL, 4.CDR + CIBIC	9	high
Kwon (2019)	Antiplatelet agents *versus* Placebo	2	12w	--	1.MMSE	4	low
Jin and Liu (2019)	Memantine *versus* Placebo	12	26w	20 mg	1.ADAS-Cog, 2.MMSE, 3.ADL, 4.CIBIC-plus orCGIC	9	high
Tan et al. (2015)	EGB761 *versus* Placebo	9	22–26w	200 mg	1.ADAS-cog, 2.CIBIC-plus orCGIC, 3.ADL	9	high
Jin and Liu (2019)	Galantamine *versus* Placebo	12	24–52w	24 mg	1.ADAS-Cog, 2.MMSE, 3.ADL, 4.CDR + CIBIC	9	high
Lopez-Arrieta and Birks (2002)	Nimodipine *versus* Placebo	12	12–24w	135 mg	1.ADAS-cog, 2.CIBIC-plus orCGIC, 3.ADL, 4.Ad	9	high
Fan et al. (2021)	NPB + Citicoline *versus* Placebo	26	12–52w	200 mg	1.MMSE, 2.BI, 3.NIHSS, 4.MoCA	9	high
Yi et al. (2020)	Olacetam *versus* Placebo	42	12w	--	1.MMSE, 2.ADL, 3.MoCA, 4.BI,5.Ad	10	high
Fan et al. (2021)	NBP *versus* Placebo	26	12–52w	200 mg	1.MMSE, 2.BI, 3.NIHSS, 4.MoCA	9	high
Fan et al. (2021)	NBP + Donepezil *versus* Placebo	26	12–52w	200mg/5 mg	1.MMSE, 2.BI, 3.NIHSS, 4.MoCA	9	high
Fan et al. (2021)	Nimodipine *versus* Placebo	26	12–52w	60 mg	1.MMSE, 2.BI, 3.NIHSS, 4.MoCA	9	high
Alvarez-Sabín et al. (2013)	Citicoline *versus* Placebo	6	12–52w	1 g	1.MMSE, 2.ADL, 3.MoCA	6	middle
Huahui and Hehua (2015)	Oxiracetam *versus* Placebo	4	24w	4.0 g	1.MMSE, 2.ADL, 3.MoCA	4	low
Ming (2016)	Salvianolate	19	12–24w	150 mg	1.NIHSS, 2.BI	10	high
Wei (2020)	TCM + western medicine *versus* Placebo	8	12–24w	--	1.MMSE, 2.MoCA, 3.ADL, 4.BI, 5.NIHSS	7	high
You et al. (2019)	Oxygen *versus* Placebo	25	12–16w	120 min, qd	1.MMSE, 2.ADL, 3.Ad	9	high
Szatmári and Whitehouse, (2009)	Vinpocetine *versus* Placebo	3	24w	30–60 mg	1.NIHSS, 2.AE	4	low

TCM, traditional chinese medicine; NBP, DL-3-n-Butylphthalide; Olacetam: Au/Polypropionic Acid Nanoparticles Loaded with Olacetam.

### MMSE score

For our search, the mini-mental state examination (MMSE) score was used to assess the effects of the medications on clinical change (Table 2). A total of 15 studies (79.0%) including Donepezil (MD: 2.21, 95% CI: −0.466 to 4.882, *p* < 0.001), MEM (MD: 1.05, 95% CI: 0.18 to 1.79, *p* < 0.001), RIV (MD: 0.32, 95% CI: −0.61 to 1.35, *p* < 0.009), Acupuncture (MD: 1.99, 95% CI: 1.09 to 2.88, *p* < 0.0001), NBP (MD: 4.89, 95% CI: 4.14 to 5.63, *p* < 0.0001), NBP + Nimodipine (MD: 2.13, 95% CI: 1.52 to 2.75, *p* < 0.0001), Citicoline (MD: 1.63, 95% CI: 1.28 to 1.98, *p* < 0.0001), NBP + Oxiracetam (MD: 1.26, 95% CI: 0.97 to 1.56, *p* < 0.0001), traditional Chinese medicine (TCM) + Western medicine (MD: 3.72, 95% CI: 0.45 to 2.15, *p* < 0.003), Oxiracetam (MD: 1.34, 95% CI: 0.88, 1.8, *p* < 0.01), oxygen (MD: 4.0, 95% CI: 3.28 to 4.73, *p* < 0.00001), and Olacetam (MD = 6.09, 95% CI: 4.55 to 7.62, *p* < 0.01) showed better outcomes for MMSE score compared to placebo. The other treatments “anti-patient agents (MD = 1.73, 95% CI: 0.91 to 3.29, *p* = 0.08)” indicated no significant difference in effectiveness as compared to placebo.

**TABLE 2:** Results of pairwise meta-analyses for the MMSE score.

Comparative	Reference medications	Number of studies	Number of controls	Number of patients	MD/OR	95% CI	I^2^	*p*
Donepezil	Placebo	7	390	396	2.21	[-0.47, 4.88]	0	0.001
MEM	Placebo	9	284	396	1.05	[0.18, 1.79]	0	0.001
RIV	Placebo	9	326	404	0.32	[-0.61, 1.35]	0	0.009
Acupuncture	Placebo	6	338	339	1.99	[1.09, 2.88]	0	0.0001
NBP	Placebo	7	264	264	4.89	[4.14, 5.63]	0	0.0001
Nimodipine	Placebo	2	32	33	2.13	[1.52, 2.75]	0	0.0001
Citicoline	Placebo	2	82	86	1.63	[1.28, 1.98]	NA	0.0001
NBP + Oxiracetam	Placebo	6	264	265	1.26	[0.97, 1.56]	0	0.0001
TCM	Placebo	6	139	141	1.30	[0.89, 6.55]	0	0.0001
Cell therapies	Placebo	4	136	137	2.80	[1.24, 4.37]	0	0.0004
Oxiracetam	Placebo	4	101	101	1.34	[0.88, 1.8]	0	0.01
Antipatient angents	Placebo	1	40	40	1.73	[0.91,3.29]	0	0.08
Oxygen	Placebo	24	931	943	4.00	[3.28, 4.73]	0	0.00001
Olacetam	Placebo	42	1864	1851	6.09	[4.55, 7.62]	0	0.01

CI, confidence interval; MD, mean difference; OR, risk ratio; I^2^, heterogeneity; MEM, memantine; RIV, rivastigmine; NBP, DL-3-n-Butylphthalide.

### NIHSS score

The National Institutes of Health Stroke scale was used to assess the effects of the medications on clinical change (Table 3). Seven studies (36.8%) showed that Citicoline (MD: −1.82, 95% CI: −2.25 to −1.40, *p* < 00001), Oxiracetam (MD: −1.15, 95% CI: −1.31, −0.98, *p* < 00001), NBP (MD: −3.86, 95% CI: 5.22 to −2.50, *p* < 0.00001), and salvianolate (MD: −2.42, 95% CI: −2.86 to −1.98, *p* < 00001) were significantly different compared with placebo. In contrast, Actovegin (MD: −0.1, 95% CI: −0.4 to 0.2, *p* < 0.455), TCM (MD: −1.45, 95% CI, −2.04 to −0.86, *p* < 0.35), and Citicoline (MD: 1.721, 95% CI: 1.065 to 2.781, *p* < 0.27) showed no change or deterioration.

**TABLE 3:** Results of pairwise meta-analyses for the NIHSS score.

Comparative	Reference medications	Number of studies	Number of controls	Number of patients	MD/OR	95% CI	I^2^	*p*
Actovegin	Placebo	6	210	220	−0.10	[-0.40, 0.20]	0	0.455
Oxiracetam	Placebo	4	324	325	−1.15	[-1.31, −0.98]	0	0.00001
NBP	Placebo	2	39	39	−3.86	[-5.22, −2.50]	0	0.00001
Salvianolate	Placebo	15	1,139	1,149	−2.42	[-2.86, −1.98]	0	0.00001
TCM	Placebo	2	70	70	−1.45	[-2.04, 0.86]	0	0.35
Citicoline	Placebo	4	172	186	−1.72	[1.065, 2.781]	0	0.027
Vinpocetine	Placebo	3	104	96	−1.40	[-2.58, −0.22]	0	0.88

CI, confidence interval; MD, mean difference; OR, risk ratio; I^2^, heterogeneity; TCM, traditional chinese medicine; NBP, DL-3-n-Butylphthalide.

### Barthel index score

The Barthel Index (BI) score was used to assess the effects of the medications on clinical change (Table 4). Eight studies (42.1%) showed that Citicoline (MD: 3.36, 95% CI, 2.80, 3.93, *p* < 0.00001), Oxiracetam (MD: 2.24, 95% CI, 0.37, 4.11, *p* < 0.032), NBP (MD: 13.53, 95% CI: 9.84, 17.22, *p* < 0.014), Donepezil (MD: 1.48, 95% CI, 1.13 to 1.83, *p* < 0.00001), salvianolate (MD: 7.68, 95% CI: 5.15∼10.21, *p* < 0.00001), TMS (MD: 9.72, 95% CI: 6.78 to 12.66, *p* < 0.00001), and Olacetam (MD = 8.71, 95% CI (17.19, 20.24), *p* <0.01) were significantly different compared with placebo. In contrast, TCM (MD: 12.36, 95% CI: 8.79 to 15.92, *p* = 0.07) and Nimodipine (MD: 2.29, HKSJ 95% CI, −17.45 to 22.03, *p* = 0.380), showed no difference compared to placebo.

**TABLE 4:** Results of pairwise meta-analyses for the BI score.

Comparative	Reference medications	Number of studies	Number of controls	Number of patients	MD/OR	95% CI	I^2^	*p*
Nimodipine	Placebo	2	76	78	2.29	[-17.45, 22.03]	0	0.38
Citicoline	Placebo	1	60	60	3.36	[2.80, 3.93]	0	0.00001
Oxiracetam	Placebo	4	172	172	2.24	[0.37, 4.11]	0	0.032
NBP	Placebo	2	88	88	13.53	[9.84, 17.22]	0	0.014
Donepezil	Placebo	2	79	79	1.48	[1.13, 1.83]	0	0.0001
Salvianolate	Placebo	11	769	773	7.69	[5.15, 10.21]	0	0.00001
TCM	Placebo	5	200	200	12.36	[8.79, 15.92]	NA	0.07
Olacetam	Placebo	5	211	208	8.71	[17.19, 20.24]	0	0.01

CI, confidence interval; MD, mean difference; OR, risk ratio; I^2^, heterogeneity; TCM, traditional chinese medicine; NBP, DL-3-n-Butylphthalide.

### ADL score

Table 5 presents the results of the comparisons of behavioral symptoms; a total of 15 studies were assessed by ADL scores. Patients treated with comparative Donepezil (MD: −0.12, 95% CI: −1.13 to 0.89, *p* < 0.0001), GAL (MD: 0.59, 95% CI: −1.60 to 2.89, *p* < 0.001), RIV (MD: 0.02, 95% CI: −1.36 to 1.40, *p* < 0.001), acupuncture (MD: 0.20, 95% CI: −3.51 to 3.91, *p* < 0.01), EGB761 (MD: −0.36, 95% CI: −0.04 to −0.28, *p* < 0.0007), Citicoline (MD: 0.15, 95% CI: 0.1 to 0.206, *p* < 0.001), NBP + Oxiracetam (MD: −2.09, 95% CI: 0.83 to 5.26, *p* < 0.001), TCM (MD: −3.07, 95% CI: −4.5 to −1.68, *p <* 0.001), Oxiracetam (MD: −1.01, 95% CI: −2.9 to 0.88, *p* < 0.01), Nimodipine (MD 0.61, 95% CI0.42 to 0.81, *p* < 0.00001), oxygen (MD: −5.91; 95% CI = −6.45, −5.36, *p* < 0.00001), tPA (OR = 0.69, 95% CI: 0.37 to 1.28, *p* < 0.041), and Citicoline (OR = 2.155, 95% CI: 1.017 to 4.566, *p* < 0.045) showed better behavioral symptoms than those administered a placebo (*p* < 0.05). Moreover, EGB761 use also improved the activities of daily living and functional outcomes (MD: 9.52; 4.66 to 14.33, *p* < 0.001). Subgroup analysis results suggest that the injectable formulation of EGB761 has more impact compared to the oral formulation. The other treatments indicated that there is no significant difference in effectiveness compared to placebo (*p* > 0.05), NBP (MD: −4.70, 95% CI: −10.94 to 1.54, *p* = 0.066), and Olacetam (MD = −3.31, 95% CI: −10.18 to 3.55, *p* = 0.34).

**TABLE 5:** Results of pairwise meta-analyses for the ADL score.

Comparative	Reference medications	Number of studies	Number of controls	Number of patients	MD/OR	95% CI	I^2^	*p*
Donepezil	Placebo	9	390	396	−0.12	[-1.13, 0.89]	0	0.0001
GAL	Placebo	9	284	396	0.59	[-1.60, 2.89]	0	0.001
RIV	Placebo	9	326	404	0.02	[-1.36, 1.40]	0	0.001
Acupuncture	Placebo	5	338	339	0.20	[-3.51, 3.91]	0	0.01
EGB761	Placebo	8	1,262	1,268	−0.36	[-0.04, −0.28]	NA	0.0007
Citicoline	Placebo	26	60	60	0.15	[0.10, 0.206]	0	0.001
NBP + Oxiracetam	Placebo	26	172	172	−2.09	[0.83, 5.26]	0	0.001
NBP	Placebo	2	79	79	−4.70	[-10.94, 1.54]	0	0.066
TCM	Placebo	2	69	71	−3.07	[-4.5, −1.68]	0	0.001
Oxiracetam	Placebo	4	101	101	−1.01	[-2.9, 0.88]	0	0.01
Nimodipine	Placebo	12	228	215	0.61	[0.42, 0.81]	0	0.0001
Oxygen	Placebo	15	454	470	−5.91	[-6.45, −5.36]	0	0.00001
Citicoline	Placebo	4	172	186	2.16	[1.02, 4.57]	0	0.0456
Olacetam	Placebo	16	828	829	−3.31	[-10.18, 3.55]	0	0.34
tPA	Placebo	3	93	211	0.69	[0.37,1.28]	0	0.041

CI, confidence interval; MD, mean difference; OR, risk ratio; I^2^, heterogeneity; GAL, galantamine; RIV, rivastigmine; NBP, DL-3-n-Butylphthalide.

### CIBIC-plus or CGIC score

The CIBIC-plus or CGIC score from the administration of other treatments was mild, whereas the CIBIC-plus or CGIC scores were significantly different between placebo groups and the following groups: Donepezil (MD: 1.07, 95% CI: 0.64 to 1.86, *p* < 0.0001), GAL (MD: 1.47, 95% CI: 0.96 to 2.34, *p* < 0.001), MEM (MD: 2.78, 95% CI: 1.05–7.29, *p* < 0.001), EGB761 (MD: 1.88, 95% CI: 1.54 to 2.29, *p* < 0.0009), and Nimodipine (MD: −0.87, 95% CI: −1.07 to −0.67, *p* < 0.00001) (Table 6).

**TABLE 6:** Results of pairwise meta-analyses for the CIBIC-plus or CGIC score.

Comparative	Reference medications	Number of studies	Number of controls	Number of patients	MD/OR	95% CI	I^2^	*p*
Donepezil	Placebo	9	390	396	1.07	[0.64, 1.86]	0	0.0001
GAL	Placebo	9	284	396	1.47	[0.96, 2.34]	0	0.001
MEM	Placebo	9	326	404	2.71	[1.05–7.29]	0	0.001
EGB761	Placebo	8	1,001	1,006	1.88	[1.54, 2.29]	NA	0.0009
Nimodipine	Placebo	12	483	487	−0.87	[-1.07, −0.67]	0	0.00001

CI, confidence interval; MD, mean difference; OR, risk ratio; I^2^, heterogeneity; MEM, memantine; GAL, galantamine.

### ADAS-cog score

For our search, the ADAS-cog score was used to assess the effects of the medications on clinical change. Seven studies (36.8%) including Donepezil (MD: −0.76, 95% CI: −2.104, 0.578, *p* < 0.001), Actovegin (MD: −3.70, 95% CI: −5.5 to −1.9, *p* < 0.001), GAL (MD: −1.67, 95% CI: −3.36 to −0.06, *p* < 0.0001), MEM (MD: −2,17, 95% CI: −3.91 to −0.53, *p* < 0.0001), RIV (MD: −0.28, 95% CI: −1.89 to 1.82, *p* < 0.0001), EGB761 (MD: −2.86, 95% CI: −3.18 to −2.54, *p* < 0.00001), and Nimodipine (MD: −7.59, 95% CI: −9.87 to −5.31, *p* < 0.0001) showed better outcomes for ADAS-cog score compared to placebo treatment (Table 7).

**TABLE 7:** Results of pairwise meta-analyses for the ADAS-cog score.

Comparative	Reference medications	Number of studies	Number of controls	Number of patients	MD/OR	95% CI	I^2^	*p*
Donepezil	Placebo	7	199	201	−0.76	[-2.10, 0.58]	0	0.001
Actovegin	Placebo	9	244	255	−3.70	[-5.5, −1.9]	0	0.001
GAL	Placebo	9	390	396	−1.67	[-3.36, −0.06]	0	0.0001
MEM	Placebo	9	284	396	−2.17	[-3.91, −0.53]	0	0.0001
RIV	Placebo	9	326	404	−0.28	[-1.89, 1.82]	0	0.0001
EGB761	Placebo	8	1,285	1,296	−2.86	[-3.18, −2.54]	NA	0.00001
Nimodipine	Placebo	12	247	243	−7.59	[-9.87, −5.31]	0	0.0001

CI, confidence interval; MD, mean difference; OR, risk ratio; I^2^, heterogeneity, MEM, memantine; GAL, galantamine; RIV, rivastigmine.

### MoCA-cog score

MoCA-cog score was observed in 18 studies. Detailed information on included studies is listed in Table 2. The clinical effect of Actovegin (MD: 1.0, 95% CI: 0.3 to 1.7, *p* < 0.03), NBP (MD: 1.05, 95% CI: 0.69 to 1.42, *p* < 0.00001), Nimodipine (MD: 0.90, 95% CI: 0.46, 1.33, *p* < 0.0001), Donepezil (MD: 1.04, 95% CI: 0.71 to 1.38, *p* < 0.00001), Oxiracetam (MD: 0.81, 95% CI: 0.62 to 1.01, *p* < 0.00001), and Oxiracetam (MD: −1.01, 95% CI: −2.9 to 0.88, *p* < 0.01) was significantly better compared with placebo treatment. Moreover, the combination use of TCM and olanzapine (MD = 4.32, 95% CI: 2.03–6.61, *p* <0.01) showed a significant increase in the overall clinical efficacy rate compared to TCM use alone (Table 8).

**TABLE 8:** Results of pairwise meta-analyses for the MoCA-cog score.

Comparative	Reference medications	Number of studies	Number of control	Number of patients	MD/OR	95% CI	I^2^	*p*
Actovegin	Placebo	6	211	219	1.00	[0.30, 1.70]	0	0.03
Acupuncture	Placebo	5	338	339	1.37	[-0.21, 2.95]	0	0.09
NBP	Placebo	7	276	281	1.05	[0.69, 1.42]	0	0.00001
Nimodipine	Placebo	9	563	558	0.90	[0.46, 1.33]	0	0.0001
Donepezil	Placebo	2	45	45	1.04	[0.71, 1.38]	0	0.00001
NBP + Oxiracetam	Placebo	2	79	79	0.81	[0.62, 1.01]	0	0.00001
TCM + Oxiracetam	TCM	2	61	67	1.73	[1.05, 2.41]	0	0.01
Oxiracetam	Placebo	4	101	101	−1.01	[-2.9, 0.88]	0	0.01

CI, confidence interval; MD, mean difference; OR, risk ratio; I^2^, heterogeneity; TCM, traditional chinese medicine; NBP, DL-3-n-Butylphthalide.

### Adverse events

The meta-analysis of Donepezil (MD: 0.15, 95% CI: 0.100 to 0.206, *p* < 0.099), Actovegin (MD: 2.09, 95% CI: 0.83 to 5.26, *p* < 0.124), GAL (MD: 5.64, 95% CI: 1.31 to 26.71, *p* < 0.31), RIV (MD: 16.8, 95% CI: 1.78 to 19.26, *p* < 0.23) vinpocetine (MD: 1.26, 95% CI: 0.71 to 2.21, *p* < 0.43), Nimodipine (MD: 0.79, 95% CI: 0.61 to 1.02, *p* < 0.61), oxygen (MD: 0.85, 95% CI: 0.26 to 2.78, *p* < 0.79), and Olacetam (OR = 0.58, 95% CI: 0.20 to 1.64, *p* = 0.30) were no significant differences in adverse events between these groups and placebo groups (*p* > 0.05) (Table 9). Among all of the trials, in the EGB761 (MD: 1.94, 95% CI: 1.51 to 2.50, *p* < 0.0007) groups, six cases of hypotension, four cases of fever, two cases of flushing, two cases of vomiting, one case of headache, one case of arrhythmia, and one case of pruritus were reported. In addition, no deaths and two serious adverse events were reported in the Actovegin group.

**TABLE 9:** Results of pairwise meta-analyses for AE.

Comparative	Reference medications	Number of studies	Number of controls	Number of patients	MD/OR	95% CI	I^2^	*p*
Donepezil	Placebo	6	284	396	0.15	[0.10, 0.21]	0	0.099
Actovegin	Placebo	9	244	255	2.09	[0.83, 5.26]	0	0.124
GAL	Placebo	9	326	404	5.64	[1.31, 26.71]	0	0.31
RIV	Placebo	9	338	339	16.80	[1.78, 19.26]	0	0.23
EGB761	Placebo	6	429	360	1.94	[1.51, 2.50]	NA	0.0007
vinpocetine	Placebo	5	213	206	1.26	[0.71, 2.21]	0	0.43
Nimodipine	Placebo	12	551	550	0.79	[0.61, 1.02]	0	0.61
Oxygen	Placebo	9	292	292	0.85	[0.26, 2.78]	0	0.79
Olacetam	Placebo	14	-	-	0.58	[0.20, 1.64]	0	0.30

CI, confidence interval; MD, mean difference; OR, risk ratio; I^2^, heterogeneity; GAL, galantamine; RIV, rivastigmine.

## Discussion

The data used in our umbrella review was from patients undergoing treatment for cognitive impairment after stroke and was used to assess the relative effectiveness and safety of these treatments. The data from published systematic reviews and meta-analyses were summarized to determine the treatment that was the most beneficial and effective for patients. Our study showed that ACEI inhibitors and NMDA antagonists, stem cell-based therapies, EGB761, and acupuncture can improve neurological deficits and activities of daily living in patients with PSCI. Antiplatelet agents (aspirin and clopidogrel), thrombolytic therapy (tPA), Oxiracetam, Citicoline, Vinpocetine, Actovegin, DL-3-n-Butylphthalide, and Nimodipine have little effect or no difference on neurological deficits or daily activities. In addition, there were no serious adverse events during treatments by ACEI inhibitors and NMDA antagonists, EGB761, and acupuncture. Interpretation of the study results requires caution to determine the best treatment strategy for patients with PSCI.

Cholinergic and neurotransmitters are vulnerable to vascular damage, which leads to cognitive impairment (Damodaran T et al., 2019). It is known that acetylcholinesterase inhibitors compensate for cerebral cholinergic neurotransmitter deficiency by inhibiting acetylcholine hydrolysis to regulate cognitive function, and that it is an effective treatment for PSCI and vascular dementia patients. The effects of ACEI inhibitors and NMDA antagonists may be considerable and there is no cure for current treatment, but other drugs that may slow the progression of PSCI patients are worth exploring. Previous studies showed that ACEI inhibitors and NMDA antagonists are beneficial for PSCI (Jongstra et al., 2016; Saini et al., 2021). In addition, one study showed that ACEI inhibitors-Donepezil showed the best performance (Kim and Kang, 2020). It is suggested by our results that neurological dysfunction and activities of daily living in people with PSCI can be improved by all ACEI inhibitors and NMDA antagonists compared to placebo treatment. Studies have shown that NMDA antagonists have led to the best-observed effects on PSCI. In our study, it was observed that NMDA antagonists treatment improved clinical effect significantly compared to placebo treatment. It is demonstrated that acupuncture has shown remarkable efficacy in PSCI (Huang et al., 2021). Our review mainly selected clinical studies to demonstrate short-term efficacy on neurological function, while PSCI is a progressive disease. Long-term clinical trials are ethically questionable, and high-quality clinical trials are critical to reveal differences in the treatment of PCSI by different treatments.

Behavioral symptoms in patients with PSCI are usually assessed by MMSE ADL/NIHSS/BI/MoCA, which assesses the severity and frequency of neuropsychiatric symptoms. Patients with PSCI progressively worsen with degrees of other disease, and the pooled data results may be affected by this. Therefore, previous meta-analysis has reported that the efficacy of stem cell-based therapies may be related to the severity of PSCI. In addition, ACEI inhibitors and NMDA antagonists and acupuncture can improve neurological dysfunction and activities of daily living in patients with PSCI. TCM was only moderate therapeutic effect on PSCI (Tan et al., 2015; Gou et al., 2020; Wen-Yue et al., 2020). In our study, Actovegin was more effective in the rate of neurological improvement compared to a placebo. However, the lack of placebo controls in NIHSS/BI score studies may result in a reduction in validity. Moreover, nimodipine can improve clinical outcomes to some extent, but it does not significantly reduce the incidence rate of adverse reactions. In addition, Donepezil affected MMSE/MoCA/ADL. Moreover, we considered treatment that showed better clinical efficacy and safety. Antiplatelet agents (aspirin and clopidogrel), thrombolytic therapy (tPA), Oxiracetam, Citicoline, Actovegin, Nimodipine, and NBP did not affect neurological deficits and daily activities due to a lack of statistical significance of the results.

Previous meta-analyses have shown that patients who were treated with cell therapies received a modest and better improvement in clinical effect. In addition, the results of both short-term and long-term analysis suggest that a combination of drugs shows a statistically significant advantage over placebo. The effect of TCM use only is not ideal (Birks and Grimley Evans, 2009; Gou et al., 2020; Kim and Kang, 2020; Wen-Yue et al., 2020), however, it is better when used in combination with Western medicine (Gou et al., 2020; Wen-Yue et al., 2020). Furthermore, antiplatelet agents (aspirin and clopidogrel), thrombolytic therapy (tPA), Oxiracetam, and NBP may play an important role in increasing the neurological function or daily activities of patients with PSCI. In this study, the AMSTAR2 scores were low for antiplatelet agents, vinpocetine, Oxiracetaman, and Citicoline in the systematic reviews analyzed, indicating that these might not be of importance to neurological function or daily activities. Further analysis is needed to elucidate the factors associated with the placebo effect increasing over time in global clinical trials.

In the treatment of PSCI, a critical issue is the safety of the treatments on a long-term basis. We extracted at least one adverse effect, such as diarrhea, nausea, gastrointestinal, cardiovascular, and other disorders. Previous meta-analyses have suggested that patioents with PSCI receiving 10 mg of donepezil (odds ratio (OR) = 3.04, 95% CI: 1.86–5.41) are at a higher risk of adverse events than those under a placebo treatment. Galantamine (OR = 5.64, 95% CI: 1.31–26.71) was associated with an increased risk of nausea. Rivastigmine (OR = 16.80, 95% CI: 1.78–319.26) was associated with an increased risk of vomiting. Moderate-certainty evidence showed that fewer people taking Memantine experienced agitation as an adverse event: RR 0.81 (95% CI 0.66–0.99) (25 fewer people per 1,000, 95% CI 1 to 44 fewer). There is also moderate-certainty evidence suggesting that Memantine is not beneficial as a treatment for agitation from three additional studies (e.g., Cohen Mansfield Agitation Inventory: clinical benefit of 0.50 CMAI points, 95% CI −3.71–4.71) (Gou et al., 2020). Moreover, it was found that statins were effective in the prevention of PSCI by actively lowering cholesterol in the study. Thrombolytic use of statins improves the overall situation, despite an increased risk of bleeding conversion. Recent studies have also linked statins to atrial fibrillation. In addition, neuroprotective drugs that promote collateral circulation may be related to the induction of vascular endothelial NO synthesis and angiogenesis (Fan et al., 2021). In addition, the rate of discontinuation due to adverse events tended to be higher in the salvianolic acid, tPA, NBP, and Nimodipine treatment groups than in placebo groups. Our study summarized that ACEI inhibitors and NMDA antagonists, stem cell-based therapies, acupuncture, and TCM plus Western medicine show no serious adverse events in patients with PSCI.

In general, the treatment for patients with PSCI is aimed at promoting independence, maintaining function, and treating symptoms. Previous meta-analyses and reviews have focused on the possible effectiveness and safety of AChEIs and NMDA antagonists (memantine) (Ebihara et al., 2007; Cao et al., 2013; Tan et al., 2015; You et al., 2019; Gou et al., 2020; Kim and Kang, 2020; Wen-Yue et al., 2020; Saini et al., 2021). As a result, we need to identify an efficacious and safe treatment paradigm for patients with PSCI. Studies have shown that ACEI inhibitors and NMDA antagonists, cell therapies, acupuncture, and Western medicine plus EGB761 improved neurological deficits and activities of daily living, and the adverse effects were mild for the treatment of PSCI. However, a larger sample size and long-term follow-up are needed to find the reliability of this treatment. Due to the efficacy of Donepezil, memantine, cell therapies, and Western medicine plus TCM in improving neurological deficits and activities of daily living, we suggest that Donepezil, memantine, and Donepezil plus TCM can be employed as first-line treatment.

## Limitations

The limitations of this study should be acknowledged. First, direct comparative evidence of treatments for patients with PSCI in our included studies was limited. Second, other factors, such as the duration and quality of studies, may have led to inconsistencies in the umbrella review. Furthermore, a considerable number of studies could not be included as they did not have the abovementioned data.

## Conclusion

Our study demonstrated that ACEI inhibitors (Donepezil) and NMDA antagonists (Memantine), EGB761, and acupuncture are the ADL and BI, MoCA, and neurological function deficit medication/therapy, respectively, for patients with PSCI. In the future, the combination of well-tolerated agents and other significant beneficial treatments should be used for patients with PSCI, which will contribute to the successful construction of a similar study.

## Data Availability

The raw data supporting the conclusion of this article will be made available by the authors, without undue reservation.
